# Enhanced In Vivo Activity of Cefditoren in Pre-Immunized Mice against Penicillin-Resistant *S. pneumoniae* (Serotypes 6B, 19F and 23F) in a Sepsis Model

**DOI:** 10.1371/journal.pone.0012041

**Published:** 2010-08-10

**Authors:** Fabio Cafini, Jose Yuste, Maria-Jose Giménez, David Sevillano, Lorenzo Aguilar, Luis Alou, Elisa Ramos-Sevillano, Martha Torrico, Natalia González, Ernesto García, Pilar Coronel, Jose Prieto

**Affiliations:** 1 Microbiology Department, School of Medicine, Universidad Complutense, Madrid, Spain; 2 Centro de Investigaciones Biológicas (CSIC) and CIBER de Enfermedades Respiratorias (CIBERES), Madrid, Spain; 3 Scientific Department, Tedec-Meiji Farma S.A., Madrid, Spain; National Institutes of Health, United States of America

## Abstract

**Background:**

Specific antibodies are likely to be present before *S. pneumoniae* infection. We explored cefditoren (CDN) total and free values of serum concentrations exceeding the MIC (t>MIC) related to efficacy in a mice sepsis model, and the effect of specific gammaglobulins on in-vitro phagocytosis and in-vivo efficacy.

**Methodology/Principal Findings:**

We used three pneumococcal isolates (serotype, MIC of CDN): Strain 1 (6B, 1 µg/ml), Strain 2 (19F, 2 µg/ml) and Strain 3 (23F, 4 µg/ml). Hyperimmune serum (HS) was obtained from mice immunized with heat-inactivated strains. In-vitro, phagocytosis by HS diluted 1/10 in presence/absence of sub-inhibitory concentrations was measured by flow cytometry including fluorescent bacteria and a neutrophil cell line. In-vivo dose-ranging experiments with HS (dilutions 1/2–1/16) and CDN (6.25 mg/kg–100 mg/kg tid for 48 h) were performed to determine the minimal protective dilution/dose (highest survival) and the non-protective highest dilution/dose (highest mortality: HS-np dilution and CDN-np dose) over 7 days. Efficacy of CDN-np in animals pre-immunized with HS-np (combined strategy) was explored and blood bacterial clearance determined. The CDN measured protein binding was 86.9%. In-vitro, CDN significantly increased phagocytosis (vs. HS 1/10). In non pre-immunized animals, t>MIC values for CDN of ≈35% (total) and ≈19% (free) were associated with 100% survival. Significant differences in survival were found between HS-np alone (≤20%) or CDN-np alone (≤20%) vs. the combined strategy (90%, 60% and 60% for Stains 1, 2 and 3), with t>MIC (total/free) of 22.8%/14.3%, 26.8%/16.0%, and 22.4%/12.7% for Strains 1, 2 and 3, respectively. Prior to the second dose (8 h), median bacterial counts were significantly lower in animals surviving vs. dead at day 7.

**Conclusions/Significance:**

In mice (CDN protein binding similar to humans) total t>MIC values of ≈35% (≈19% free) were efficacious, with a decrease in the required values in pre-immunized animals. This reinforces that immunoprotection to overcome resistance may provide lifesaving strategies.

## Introduction

Differences between in vitro activity and in vivo efficacy of antimicrobials may result from the participation of the immune system in bacterial eradication and/or the limitation of antibiotic activity by the binding of antibiotics to serum proteins (protein binding). Evidence shows that the successful outcome of infections caused by *Streptococcus pneumoniae* in humans depends on the humoral arm of the immune system (since opsonin/antibody-dependent phagocytosis is the major defence mechanism against *S. pneumoniae*) and on treatment with an adequate antibiotic. Natural defences and antibiotics may act concomitantly when using drugs as β-lactams acting on the cell wall that anchors the capsule. Since colonisation is to some extend a B-cell immunising event [1], [2] and preventive measures as pneumococcal vaccination are increasingly being used, antibodies to capsular polysaccharides (a surrogate marker of immunity) are likely to appear before infection. In this situation, the appearance of pneumococcal sepsis indicates defective protection against pneumococcal invasion.

All these facts were explored in previous mice sepsis models (using a serotype 6B isolate non-susceptible to penicillin as infecting strain) where bacteremia with high colony counts was related with mortality, and both (bacteremia and mortality) decreased in a dose-related trend by administration of hyperimmune serum [3] or β-lactams (amoxicillin or cefotaxime) [4]. When combined therapies (hyperimmune serum plus β-lactam) were explored, the strategy based on passive immunisation (prior to infection) followed by β-lactam administration post-infection was more effective than serotherapy plus antibiotherapy post-infection [5]. Pharmacodynamically, passive pre-immunisation of animals resulted in a significant reduction in the value of the parameter predicting efficacy for β-lactams, i.e, the time (% dosing interval) that serum concentrations exceed the MIC (t>MIC) [6] when total drug was considered.

Despite pharmacokinetic differences between mice and humans, animal models have been critical to our understanding of pharmacokinetic/pharmacodynamic (PK/PD) relationships to estimate which PK/PD parameter (t>MIC, AUC/MIC, Cmax/MIC…) is the best predictor and the adequate magnitude of the parameter. However, antibiotics with high protein binding require special consideration since, classically, it is accepted that only the unbound fraction of the compound is active in vitro and presumably in vivo; the different protein binding rate in mice and humans limiting the extrapolation of results for high protein binding antibiotics.

Characterization of interactions between antibiotics and serum proteins is essential in the assessment of pharmacodynamic implications on antibacterial activity. The aims of this study, using *S. pneumoniae* isolates from different serotypes (6B, 19F and 23F) with increasing MIC values, were to explore for cefditoren (controlled with ceftriaxone for serotype 19F): a) Values of t>MIC (total and free) related to efficacy in a mice sepsis model, taking advantage of the similar high protein binding rate of cefditoren (88%) in human and mice sera [7], [8], b) The in vitro effect of sub-inhibitory concentrations and specific antibodies on the interaction of *S. pneumoniae* and phagocytes, and c) The in vivo effect of the presence of specific antibodies to *S. pneumoniae* (passive immunization) on t>MIC values (total and free) related to therapeutic efficacy in the mice sepsis model.

## Results

### Mice immunized with inactivated pneumococcal strains produced antigen-specific serum antibodies

To evaluate the systemic humoral immune response to the corresponding immunogenic suspension with the different strains of the study, the levels of serum antigen-specific antibodies generated by immunized mice, were compared to those of control (non- immunized) mice. Antibody levels were very low in the control group (59 µg/ml) whereas those obtained in the immunized groups were serotype-specific with the immunogenic suspension against the serotype 6B strain rendering the highest levels of specific antibodies. Actually, overall IgG levels determined in hyperimmune sera were 1056 µg/ml in animals immunized against Strain 1 (serotype 6B), 371 µg/ml in those immunized against Strain 2 (serotype 19F), and 251 µg/ml in those immunized against Strain 3 (serotype 23F).

These results showed that immunized mice generated target-specific antibodies in response to intraperitoneal immunization with the different heat killed bacterial suspensions.

### Cefditoren enhances opsonophagocytosis of *S. pneumoniae* in the presence of specific antibodies

**Figure 1:**
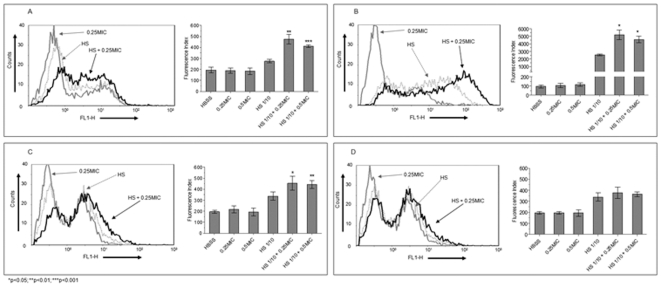
In vitro opsonophagocytosis using HL-60 cell line. Flow cytometry histograms and the corresponding fluorescence indices showing opsonophagocytosis for Strain 1 (A), Strain 3 (B), and Strain 2 with cefditoren (C) and ceftriaxone (D).

**Figure 2:**
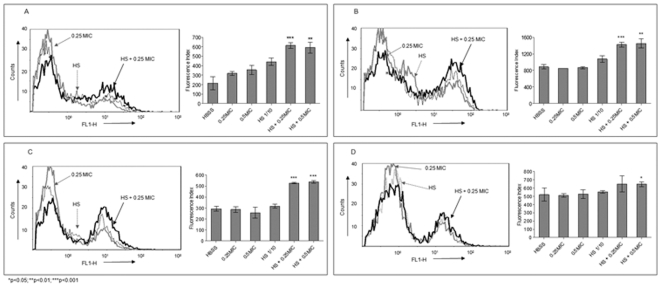
In vitro opsonophagocytosis using fresh mice neutrophils. Flow cytometry histograms and the corresponding fluorescence indices showing opsonophagocytosis for Strain 1 (A), Strain 3 (B), and Strain 2 with cefditoren (C) and ceftriaxone (D).

To explore whether subinhibitory concentrations of cefditoren could increase the phagocytosis of *S. pneumoniae* by neutrophils in the presence of specific antibodies, a flow cytometry assay was performed. In phagocytosis assays measured by flow cytometry it is important to analyse not only the percentage of positive cells but also the amount of bacteria phagocytosed per neutrophil. To address both parameters, results were expressed as a Fluorescence Index (calculated as the geometric mean of the fluorescence multiplied by the percentage of positive cells). The incubation of pneumococcal strains with different subinhibitory concentrations of cefditoren or ceftriaxone did not significantly (p>0.05) increase phagocytosis compared to control samples that were incubated in Hanks' balanced salt solution (HBSS), which measure the level of phagocytosis by the neutrophil itself in the absence of opsonins or any other mediator (Fig. 1 and 2). The incubation of the different strains with the corresponding hyperimmune serum (dilution 1/10) increased the uptake of *S. pneumoniae* in experiments using HL-60 cells with all strains (Fig. 1) and in those using mice neutrophils with Strains 1 and 3 (Fig. 2). In addition, the incubation of *S. pneumoniae* with subinhibitory concentrations of cefditoren in the presence of specific antibodies significantly increased the phagocytosis against the three strains compared to the phagocytosis mediated by the 1/10 dilution of hyperimmune serum or the subinhibitory antibiotic concentrations independently, both using HL-60 (p<0.03) (Fig. 1A–C) and mice neutrophils (p≤0.001) (Fig. 2A–C). This was not the case of ceftriaxone and Strain 2 where the phagocytosis mediated by HL-60 cells was not statistically different (p = 0.2) from the one mediated by the 1/10 dilution of hyperimmune serum itself (Fig. 1D), although in experiments with mice neutrophils the phagocytosis with 0.25× MIC was significant (p = 0.012) but not with 0.5× MIC (p = 0.1) (Fig. 2D). Overall, these results suggest that in the presence of specific antibodies to *S. pneumoniae*, the addition of subinhibitory concentrations of cefditoren increased the efficiency of phagocytosis mediated by professional phagocytes.

### Efficacy obtained with antibiotic treatment is improved in the presence of specific antibodies

Survival experiments were performed using a mice sepsis model of infection. Minimal lethal doses, producing 100% mortality rates over a 7-day follow-up period, were 3.5×10^7^ cfu/ml for Strain 1, 1.6×10^7^ cfu/ml for Strain 2 and 4.7×10^6^ cfu/ml for Strain 3.

To explore the protective capacity of the hyperimmune sera, groups of animals were immunized with different dilutions of the sera 1 h before challenge with a lethal dose of the corresponding bacteria and survival was followed for 1 week. In passively immunized animals (with the 1/8 or 1/4 dilutions) that did not receive antibiotic treatment, the survival rate was 0% in animals infected with Strains 1 and 3 and 20% in those inoculated with Strain 2. With the 1/2 dilution, percentages of survival were 40% for Strains 1 and 2, and 20% for Strain 3. Therefore, the first dilution showing 100% (or the highest) mortality, that was considered the non-protecting dilution of hyperimmune serum (HS-np), was 1/4 for Strains 1 and 2 and 1/2 for Strain 3 (Tables 1 and 2).

**Table 1:** Pharmacodymanics and efficacy against Strains 1 and 3.

	Strain 1 (serotype 6B; MIC_CDN_ = 1 µg/ml)			Strain 3 (serotype 23F; MIC_CDN_ = 4 µg/ml)		
Experimental group	t>MIC	*f*t/MIC	% Survival at day 7	t>MIC	*f*t/MIC	% Survival at day 7
**CDN (mg/kg)**						
Control	-	-	0	-	-	0
6.25	21.6	13.1	0	15.8	4.9	ND
12.5	22.8	14.3	0	17.2	8.1	20
25	31.7	17.4	60	20.8	10.5	20
50	34.6	19.3	100	22.4	12.7	20
100	45.0	22.7	ND	27.3	15.8	60
**HS**						
Control	-	-	0	-	-	0
HS 1/4	-	-	0	-	-	0
HS 1/2	-	-	40	-	-	20
**HS + CDN (mg/kg)**						
Control	-	-	0	-	-	0
CDN^a^	22.8	14.3	0	22.4	12.7	10
HS^b^	-	-	0	-	-	10
HS^b^ + CDN^a^	22.8	14.3	90	22.4	12.7	60

a 12.5 mg/kg for strain 1 and 50 mg/kg for strain 3

b 1/4 dilution for strain 1 and 1/2 for strain 3

t>MIC, *f*t>MIC and survival at day 7 in animals infected with strains 1 and 3 obtained in experiments with dose-ranging cefditoren (CDN), dose-ranging hyperimmune serum, and in experiments administering CDN in pre-immunized animals

**Table 2:** Pharmacodymanics and efficacy against Strain 2.

	Cefditoren (MIC = 2 µg/ml)			Ceftriaxone (MIC = 4 µg/ml)		
Experimental group	t>MIC	*f*t/MIC	% Survival at day 7	t>MIC	*f*t/MIC	% Survival at day 7
**Antibiotic (mg/kg)**						
Control	-	-	0	-	-	0
6.25	18.7	9.6	0	20.6	0.0	0
12.5	19.9	11.3	20	26.9	5.4	80
25	24.5	13.7	0	41.1	8.7	100
50	26.8	16.0	0	45.6	11.9	100
100	36.1	19.2	100			ND
**HS**						
Control	-	-	0	-	-	0
HS 1/4	-	-	20	-	-	20
HS 1/2	-	-	40	-	-	40
**HS + Antibiotic (mg/kg)**						
Control	-	-	0	-	-	0
Antibiotic^a^	26.8	16.0	0	20.6	0.0	0
HS^b^	-	-	10	-	-	0
HS^b^ + Antibiotic^a^	26.8	16.0	60	20.6	0.0	20

a 50 mg/kg for cefditoren and 6.25 mg/kg for ceftriaxone

b 1/4 dilution

t>MIC, *f*t>MIC and survival at day 7 in animals infected with strain 2 (serotype 19F) obtained in experiments with dose-ranging cefditoren and ceftriaxone, dose-ranging hyperimmune serum, and in experiments administering antibiotics in pre-immunized animals

The protection conferred by the antibiotics of the study was investigated in the sepsis model. Groups of mice were challenged with a lethal dose of the corresponding strain and 1 h later antibiotic treatment was initiated, with administration at 8 h intervals for a total of 6 doses. Treatment with 50 mg/kg of cefditoren in animals infected with Strain 1 or 100 mg/kg in animals infected with Strain 2 produced 100% survival rates, while in those infected with Strain 3, the 100 mg/kg regimen only produced 60% survival (Tables 1 and 2). The highest cefditoren doses that produced the lowest survival rate (i.e, antibiotic non-protective doses - A-np) were 12.5 mg/kg for Strain 1 (0% survival) and 50 mg/kg for Strain 2 (0% survival) and 3 (20% survival). In the case of ceftriaxone and Strain 2 (MIC = 4 µg/ml), 100% survival was obtained with 25 mg/kg, while 0% survival was obtained with the 6.25 mg/kg dose (A-np) (Table 2).

**Figure 3:**
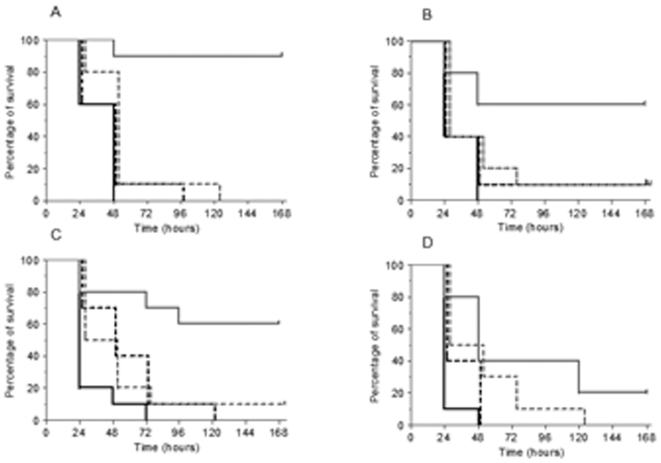
Therapeutic efficacy. Survival curves. Lines: continuous black (control), dotted black (antibiotic non-protective dose; A-np), dotted grey (hyperimmune serum non-protective dilution; HS-np) and continuous grey (A-np treatment in animals pre-immunized with HS-np). (A) Strain 1 (A-np: 12.5 mg/kg; HS-np = 1/4), (B) Strain 3 (A-np: 50 mg/kg; HS-np = 1/2), (C) Strain 2 with cefditoren (A-np: 50 mg/kg; HS-np = 1/4), and (D) Strain 2 with ceftriaxone (A-np: 6.25 mg/kg; HS-np = 1/4).

To investigate the possibility of enhanced survival by the antibiotic treatment in the presence of specific antibodies, group of animals previously immunized with the Hs-np dilution and challenged 1 h later with the lethal dose of the corresponding bacteria were treated at 8 h intervals with the A-np for each strain. Survival rates in mice infected with Strain 1 increased from 0% in animals receiving either the HS-np or the A-np alone to 90% in animals receiving the combination. In mice infected with Strain 2, survival rates raised from 0% (A-np alone) or 10% (HS-np alone) to 60% (HS-np+A-np), whereas in those infected with Strain 3, survivals changed from 10% (A-np alone or HS-np alone) to 60% (HS-np+A-np) (Tables 1 and 2). In the case of ceftriaxone and Strain 2, survival rates increased from 0% to 20% (Table 2). Significant differences were found between Kaplan-Meier survival rates obtained with the combined strategy vs. HS-np alone or A-np alone for cefditoren and Strain 1 (p<0.0001), Strain 2 (p≤0.01) and Strain 3 (p≤0.05), but not for ceftriaxone and Strain 2 (p = 0.1) (Figure 3). These results confirm that the efficacy of cefditoren treatment against the non-susceptible strains was markedly higher in the presence of specific antibodies to *S. pneumoniae*.

### Pre-immunization results in a reduction in the values of pharmacodynamic parameters predicting efficacy

Pharmacokinetic and pharmacodynamic parameters for cefditoren and ceftriaxone were calculated with concentrations experimentally measured in mice sera (Table 3). Rates of protein binding in mice sera were 86.9% for cefditoren and 91.9% for ceftriaxone. In addition, t>MIC (total) and *f*t>MIC (free) were calculated and related with percentages of survival at day 7 in mice receiving the antibiotic (alone) or hyperimmune serum+antibiotic (Tables 1 and 2). In non-pre-immunized animals, t>MIC values for cefditoren of ≈35% for total drug (corresponding to ≈19% for free drug) were associated with 100% survival (occurring with 50 mg/kg cefditoren for Strain 1, MIC = 1 µg/ml, and with 100 mg/kg for Strain 2, MIC = 2 µg/ml). Lower t>MIC values (of 27.3% and 31.7% for total drug corresponding to 15.8% and 17.4% for free drug, respectively) were associated with 60% survival, occurring with 25 mg/kg cefditoren for Strain 1 (MIC = 1 µg/ml) and 100 mg/kg for Strain 3 (MIC = 4 µg/ml), respectively. When t>MIC values for cefditoren were lower than 26.8% for total drug (16.0% for free drug) survivals were <20% (12.5 mg/kg for Strain 1 or 50 mg/kg for Strains 2 and 3) (Tables 1 and 2).

**Table 3:** Pharmacokinetics.

	C_0_ (µg/ml)	t1/2 (h)	AUC_last_ (µg× h/ml)	T_last_ (h)
**Cefditoren (mg/kg)**				
**6.25**	53.0	0.9	30.4	6
**12.5**	168.4	1.1	64.1	8
**25**	232.5	0.9	101.3	8
**50**	290.6	1.1	124.4	8
**Ceftriaxone (mg/kg)**				
**6.25**	28.6	0.9	24.0	2
**12.5**	101.2	0.9	54.8	4
**25**	483.7	0.9	158.8	6

Pharmacokinetic parameters of cefditoren and ceftriaxone from serum concentrations experimentally determined in healthy animals

In the case of ceftriaxone and Strain 2 (MIC = 4 µg/ml), 100% survival was obtained with t>MIC of 41.1% for total drug (8.7% for free drug) corresponding to a ceftriaxone 25 mg/kg administration, while 0% survival was obtained with t>MIC of 20.6% for total drug (0% free drug) corresponding to 6.25 mg/kg ceftriaxone administration.

However, cefditoren t>MIC values linked to high survival rates markedly decreased in pre-immunized animals and therefore with specific antibodies in the systemic circulation: from ≈35% (≈19% free) to ≈25% (≈15% free) for Strains 1 and 2, and from 27.3% (15.8% free) to 22.4% (12.7% free) for Strain 3. Overall, cefditoren t>MIC values linked to survival (90% for Strain 1 and 60% for Strains 2 and 3) in pre-immunized animals with the HS-np dilution were associated with <10% survival rates in non-immunized animals. This was not the case of ceftriaxone where t>MIC values with the A-np dose were linked to survivals ≤20%, regardless the pre-immunization or not of animals with the HS-np dilution.

### Early bacterial clearance of *S. pneumoniae* from the bloodstream is associated with survival

To study the relationship between survival/mortality with rapid bacterial clearance from blood, animals were grouped by outcome at day 7 (one group for surviving animals and one for those dead at day 7), regardless the study arm (control, HS-np, A-np or HS-np+A-np). Median blood colony counts (cfu) per ml (cfu/ml) at time 8 h (when all animals were still alive and prior to the administration of second antibiotic dose) for the three strains were significantly lower in animals surviving at day 7 than in those dying from 8 h on: 8.25×10^4^ vs. 1.20×10^6^ for Strain 1 (serotype 6B), 1.14×10^5^ vs. 5.59×10^8^ for Strain 2 (serotype 19F) and 1.25×10^4^ vs. 3.55×10^6^ for Strain 3 (serotype 23F). These results confirm that rapid reduction in bacterial load conditions therapeutic outcomes, and that strategies associated with lower mortality had a translation in rapid bacterial clearance.

## Discussion

Preventive and therapeutic measures against *S. pneumoniae* as vaccination and antibiotic therapy have modified not only the disease burden but also resistances in this bacterial species. It is well known that in invasive isolates penicillin/erythromycin non-susceptibility is clustered in certain serotypes. The 7-valent conjugate pneumococcal vaccine (PCV7), including serotypes most associated to penicillin/erythromycin non-susceptibility, was introduced for children immunization in 2000 s, impacting in the current decade the prevalence of the different serotypes and reversing the increasing prevalence of penicillin non-susceptibility in *S. pneumoniae* in previous decades, with a herd effect in adults [9], [10].

The strains used in the present study were selected, among strains belonging to serotypes included in currently available pneumococcal vaccines, based on their high penicillin MIC (the lower the in vitro activity of the antibiotic, the greater the chance to evidence the potential in vivo synergism between antibiotic serum concentrations and immunoglobulins present by passive immunization). For this reason, MICs of penicillin, cefditoren and ceftriaxone for the three study strains are higher than MIC_90_ values of the three compounds in published surveillance studies [11], [12]. The strains of this study belonged to serotypes 6B, 19F and 23F, thus synergism was explored against serotypes included in immunization campaigns. In a recent in vitro study analyzing clinical isolates that exhibited very high penicillin, cefotaxime and amoxicillin resistance (MIC≥16 µg/ml) associated with resistance to all oral β-lactams and macrolides, all isolates belonged to serotypes 19F and 23F [13], [14]. In addition, isolation of serotypes 6B, 19F and 23F (among PCV7 serotypes) has been identified as an independent risk factor for 30-day mortality in bacteremic disease in a recently published study [15]. It is in this context, with troublesome strains, where therapeutic strategies present added value since these strains are a challenge for antibiotic treatment.

In vivo, antibiotics interact with bacteria in a more complex way than in vitro due to the presence of serum proteins acting as an interface. While specific immunoglobulins and complement may enhance antibacterial activity of β-lactams, albumin may limitate its activity in highly bound agents. Apart from exploring combined effects of antibiotics and the immune system, the study arms consisting in antibiotic administration alone (in non-immunized animals) provided interesting pharmacodynamic information on cefditoren and ceftriaxone, considering their similar protein binding rates in mice and humans. An in vitro approach to the influence of protein binding on the cefditoren activity was performed in a previous in vitro pharmacodynamic simulation over 24 h of serum concentrations (achieved after 400 mg cefditoren bid administration) using broth containing physiological concentrations of human albumin (4 g/dl) against four *S. pneumoniae* isolates exhibiting MICs of 0.25 and 0.5 µg/ml [16]. In that study, reductions in the initial inocula were >99.9% for the strain with MIC = 0.25 µg/ml and from 52.9% to 96.8% for the strain with MIC = 0.5 µg/ml, with free t>MIC values ≤24% (experimental protein binding of 87.1%) [16]. In the present animal model, total t>MIC of ≈35% (*f*t>MIC values of 20%) were associated with therapeutic efficacy of cefditoren (with an experimentally measured binding rate of 86.9% in mice serum), using as infecting strains isolates with cefditoren MICs of 1, 2 or 4 µg/ml. Monte Carlo simulations have estimated that serum concentrations of cefditoren after 400 mg bid administration in humans exceed MICs of 1 µg/ml for 44.1% of the dosing interval [17]; the results of the present model suggesting the appropriateness of this t>MIC value for pharmacodynamic efficacy of cefditoren.

Animal models, as pragmatic studies, have been critical to our understanding of PK/PD relationships to estimate which pharmacodynamic parameter is the best predictor (neutropenic animal models) and the magnitude of this parameter (immunocompetent animal models) [18]. However explicative animal models are needed in the case of *S. pneumoniae* since, as previously commented, antibodies to capsular polysaccharides are likely to be present before infection because colonization is an immunizing event and preventive measures as vaccination are increasingly being used. Previous animal models using a serotype 6B isolate as infecting strain showed the lower t>MIC value (total drug) associated with 100% survival in immunized vs. non-immunized animals [5] for amoxicillin and cefotaxime. If synergism could be expected for other β-lactams or other serotypes remained to be explored. The benefit of the synergism would be not the antibiotic dose decrease but the coverage of strains with high MIC. In the current model previous passive immunization of animals resulted in a decrease in t>MIC values for cefditoren associated with efficacy against the three study strains belonging to serotypes 6B, 19F and 23F. While in immunized animals t>MIC values of 22.8% (total) and 14% (free) for cefditoren were associated with 90% survival for Strain 1 and 60% survival (with similar t>MIC values) for Strains 2 and 3, these t>MIC values were associated with survivals <10% in non-immunized animals (absence of specific antibodies). However this was not observed for ceftriaxone where no synergism (or even more collaboration) of ceftriaxone and specific antibiotics was demonstrated (total t>MIC values of 20.6% were not efficacious regardless the presence or not of specific antibodies). A possible explanation for this could be that all ceftriaxone binds serum proteins under the combined treatment conditions implying that no free ceftriaxone was available (negligible free t>MIC for total t>MIC values of 20.6%) for the potential enhanced phagocytosis and protection. In addition in vitro opsonophagocytosis experiments showed that the collaboration of specific antibodies and cefditoren was markedly higher than the one found with ceftriaxone against Strain 2 when using mice neutrophils, with a lack of collaboration (for ceftriaxone) when using HL-60 cells. Subinhibitory concentrations of cefditoren significantly enhanced the phagocytosis produced by the hyperimmune serum (1/10 dilution) against the three serotypes tested regardless the source of neutrophils used. For extracellular capsulated pathogens such as *S. pneumoniae*, phagocytosis is thought to be an important immune mechanism for controlling infection [19], [20]. Although there has been considerable emphasis on the role of anticapsular antibodies for immunity to *S. pneumoniae* in vaccinated individuals, complement and antibodies to non-capsular antigens are likely to be important [21]. The present study was performed with hyperimmune serum obtained by mice immunisation with heat-inactivated whole cell *S. pneumoniae*. Our results confirmed that phagocytosis mediated by professional phagocytes was increased in the presence of subinhibitory concentrations of cefditoren and specific antibodies to *S. pneumoniae* (in a higher extend in experiments with fresh mice neutrophils).

The objective of antibiotic treatment is the rapid decrease of bacterial load. Recently, bacterial load in patients with pneumococcal pneumonia has been associated with failure (likelihood of death) [22]. Previous animal models showed the effect of specific antibodies or β-lactams (amoxicillin and cefotaxime) on the bacteremic profile over 7 days and its relation with mortality [3], [4]. In the present study the bacteremic profile was also related with mortality (data not shown), but we further investigated mortality prediction by means of relating bacterial counts in blood in the 8 h-period after the first dose administration with mortality from 8 h on, as a marker of the importance of rapid clearance of bacteria (in vivo bactericidal activity) on the therapeutic outcome. At 8 h (prior to the second dose administration), when all animals were still alive (including controls), the group of animals dead at day 7 (regardless the day of death) showed high median bacterial counts (≥1.20×10^6^ cfu/ml) whereas in the group of surviving animals at day 7, median bacterial counts were lower (≤1.14×10^5^ cfu/ml). This stresses the importance of rapid antibacterial activity producing early reduction of the bacterial load for therapeutic efficacy.

The results of this study show that in mice, where the protein binding rate is similar to the one in humans, cefditoren total t>MIC values of ≈35% were linked to therapeutic efficacy, and the required values decreased when animals were pre-immunized. Early clearance of microorganisms from blood (rapid antibacterial activity) was associated with survival. The combined effects of cefditoren and hyperimmune serum against the three strains from serotypes 6B, 19F and 23F reinforce the idea that the development of immunoprotection to overcome resistance may potentially provide new lifesaving strategies [23].

## Materials and Methods

### Strains

Three *S. pneumoniae* strains were used throughout the study: Strain 1 (serotype 6B; penicillin MIC = 2 µg/ml, cefditoren MIC = 1 µg/ml), Strain 2 (serotype 19F; penicillin MIC = 1 µg/ml, cefditoren MIC = 2 µg/ml, ceftriaxone MIC = 4 µg/ml), and Strain 3 (serotype 23F; penicillin MIC = 16 µg/ml, cefditoren MIC = 4 µg/ml). Microorganisms were grown in Todd-Hewitt broth supplemented with 0.5% yeast extract (Difco, Detroit, Mich.), aliquoted, and stored at −80°C in Todd-Hewitt broth with 10% glycerol. These bacterial aliquots were used in all the following experiments.

### Antibiotics

Powers of known potency of cefditoren (Tedec-Meiji Farma S.A, Madrid, Spain) and ceftriaxone (Sigma-Aldrich Chemical Co., St Louis, MO) were used in experiments carried out with the three strains in the case of cefditoren and Strain 2 in the case of ceftriaxone.

### Animals

BALB/c female mice aged from 8 to 12 weeks weighing 19 to 22 g were purchased from Harlan Laboratories Models (Barcelona, Spain).

### Ethics Statement

Prevailing regulations regarding the care and use of laboratory animals in Spain and the European Community were followed throughout the study. The protocol of the study was approved by the Ethical Committee for Animal Experimentation of Universidad Complutense, Madrid (Spain) (Approval certificate issued on February 20th, 2009).

### Hyperimmune serum

Hyperimmune serum from the different strains was obtained as previously described [6]. Briefly, bacteria in the logarithmic phase of growth were inactivated by heat treatment at 60°C for 1 h, confirming the absence of live bacteria in the immunogenic suspension by plating a small volume onto blood agar plates. The antigenic suspension was kept at −70°C as small aliquots for further inoculations. Groups of 20 mice were inoculated weekly (up to five weeks) by the intraperitoneal route, with 200 µl of the inactivated bacterial suspension containing 10^7^ cfu/ml of the corresponding strain. The animals were exsanguinated by cardiac puncture to obtain the serum. Levels of specific IgG antibodies to capsular serotypes 6B, 19F and 23F were determined for both the pre-immune and immune sera by using the Nab IgG Plus Spin Column (Pierce®, Rockford, USA) and a Bradford dye protein assay [24]. Hyperimmune sera were aliquoted and stored at -70°C for further use both in the in vitro and in vivo studies (see below).

### Opsonophagocytosis

To explore the effect of combining sub-inhibitory antibiotic concentrations and specific antibodies on the interaction of *S. pneumoniae* with phagocytes, the level of phagocytosis was measured by using a previously described flow cytometry opsonophagocytic assay including fluorescent bacteria and a neutrophil cell line [25], [26]. In addition the assay was performed using fresh neutrophils isolated from mice peripheral blood following previously described methods [27]. *S. pneumoniae* strains were fluorescently labelled by incubation with 5,6-carboxyfluorescein-succinimidyl ester (FAM-SE; Molecular Probes, Eugene, OR) solution (10 mg/ml in dimethyl sulfoxide; Sigma) in 0.1 M sodium bicarbonate buffer for 1 h at 37°C, washed five times with Hanks balanced salt solution (HBSS) and stored in aliquots at −70°C in 10% glycerol (approximately 10^9^ cfu/ml) for further use. The cell line HL-60 (CCL240; American Type Culture Collection, Rockville, Maryland) was used to provide the effector cells after differentiation into granulocytes by using previously described protocols [25], [26], [28]. Differentiation was confirmed before the assays using a monoclonal antibody to CD11b (kindly supplied by Dr. C. Bernabeu, CIB-CSIC) which is a useful marker of granulocytic differentiation [29]. HL-60 cells were harvested by centrifugation and washed twice with HBSS and once with HBSS in the presence of calcium and magnesium ions. FAM-SE labeled bacteria (10^6^ cfu) were opsonized with hyperimmune serum diluted 1/10 in the presence or absence of sub-inhibitory concentrations (0.25×or 0.5×MIC) of cefditoren (Strains 1, 2 and 3) or ceftriaxone (Strain 2) for 1 h at 37°C with 150 rpm shaking. Negative controls, using the same volume of HBSS, were also included [25], [26]. HL-60 cells (10^5^ cfu/well) were added to opsonized bacteria in a microtiter plate and incubated for 30 minutes at 37°C with shaking, after which the bacteria and cells were fixed using 3% paraformaldehyde and analyzed using a Cytomics FC500 Beckman Coulter flow cytometer (Beckman Coulter, Miami, USA). A minimum of 6000 cells per sample were analyzed. Results were expressed as fluorescence index defined as the proportion of positive neutrophils for fluorescent bacteria multiplied by the geometric mean fluorescence intensity (MFI) which correlates with the amount of bacteria phagocytosed per cell [26], [30]. Control experiments with strain no. 2, pre- and post- opsonization confirmed that bacterial fluorescence was not affected by opsonization with serum or by the presence of antibiotics (data not shown).

The two-tailed Student's t-test was used to compare fluorescence indices obtained with the different experimental groups.

### Animal model

#### Determination of the minimal lethal dose

Groups of 5 mice per dilution were inoculated intraperitoneally (i.p.) with different inocula in Todd-Hewitt broth: 1×10^6^, 5×10^6^, 1×10^7^, 5×10^7^, 1×10^8^ cfu/ml (spectrophotometrically measured) to determine the minimal dose that produced a 100% mortality rate over a 7-day follow-up period (i.e., the minimal lethal dose).

#### Determination of protection by hyperimmune serum

Groups of five mice per dilution were inoculated i.p. with 200 µl of serial doubling dilutions of hyperimmune serum ranging from one-half to one-eighth. The animals included in the control group were injected with a placebo (phosphate buffer saline, PBS). After 1 h, the mice received one lethal dose of bacteria by the i.p. route. The animals were observed for 7 days. The first dilution showing 100% (or the highest) mortality was considered the non-protecting dilution of hyperimmune serum (HS-np).

#### Determination of protection by antibiotics

Groups of five mice per antibiotic dose were infected i.p. with one lethal dose of bacteria. After 1 h, animals were treated three times a day for 48 h by administering 100 µl by the subcutaneous route in a dose-ranging study, with the doses ranging from 6.25 to 100 mg/kg of body weight. The animals included in the control group received PBS. The animals were observed, and the numbers of deaths were recorded for 7 days.

The highest dose that produced 0% (or the lowest) survival rate was considered the non-protective dose (A-np).

#### Determination of antibiotic protection in the presence of antibodies

Groups of 10 animals received a single i.p. dose of hyperimmune serum (using the HS-np dilution) administered 1 h prior to i.p. inoculation of the bacterial minimal lethal dose. Antibiotic treatment with the A-np dose was initiated 1 h after the pneumococal challenge and was continued every 8 h, with a total of six subcutaneous doses being administered. The animals were observed, and the numbers of deaths were recorded for 7 days. On each day of inoculation, three control groups were used: one received placebo, one received the HS-np alone and one received the A-np regimen alone.

An ordinal log-rank (Mantel-Cox) test was used to compare survivals in experimental groups.

#### Determination of bacterial counts in serum

Bacteremic profiles (colony counts in blood) were determined in the groups of 10 animals receiving the combined HS-np+A-np treatment as well as in the three control groups mentioned above. To this end, five animals per group were randomly chosen and blood samples were collected at 2, 4, 6, 8, 24, 48, 72, 96, 120, 144 and 168 h post-infection (only from live animals). To collect blood samples, tails were disinfected and anaesthetized (local anaesthesia; ethyl chloride, Cloretilo Cheminosa, Ern, Barcelona, Spain), and the terminal portion of the tail was eliminated with scissors. An 8-µl blood sample was collected by pressing the tail, resuspended in Todd-Hewitt broth containing 50 units of heparin (Calbiochem, Darmstadt, Germany), and plated onto blood agar for colony counting. To obtain the subsequent blood samples, the crust was removed and again, by pressing the anaesthetized tail, an 8-µl blood sample was collected and processed. This procedure ensured that blood collection did not compromise mouse survivability as previously demonstrated [3], [4]. The lower limit of detection was 10^2^ cfu/ml.

#### Determination of antibiotic concentrations in serum

Concentrations in serum were determined in healthy animals after administration of a single subcutaneous dose of all antibiotic doses tested in the antibiotic dose-ranging studies. Blood samples were collected at 0.25, 0.5 1, 2, 4, 6, and 8 h after dosing from groups of two animals per dose, timepoint and antibiotic. Concentrations were measured by bioassay with *Morganella morganii* ATCC 8076H for cefditoren and *Escherichia coli* NCTC 10418 for ceftriaxone as reference organisms.

#### Pharmacokinetic analysis

Protein binding in mice serum was measured by an ultrafiltration method described by Craig and Suh [31] for cefditoren and ceftriaxone concentrations of 4 µg/ml and 64 µg/ml, respectively, by using a centrifugal system device (Centrifree®, Amicon Bioseparations, Millipore, Tullagreen, Ireland). Antibiotic concentrations in mice serum (pre-filtered samples) and in ultrafiltrates recovered in the polyethylene filtrate cups were measured by bioassay (see above). Percentages of antibiotics bound to mice serum proteins were calculated using the expression:

[antibiotic in serum]-[antibiotic in ultrafiltrates]**/**[antibiotic in serum]×100.

Experimentally determined values of protein binding in mice serum was used for calculating free concentrations of each antibiotic.

Total and free concentrations for each antibiotic were analyzed by a noncompartmental approach with the 5.2 Win-Nonlin program (Pharsight, Mountain View, CA.). The theoretical concentration at time zero (obtained by back extrapolation to the origin of the elimination regression line) and t1/2 was calculated by least-squares non-lineal regression analysis. The area under the serum concentration-time curves (AUCs) from time zero to infinity was calculated by the trapezoidal rule. t>MIC and free t>MIC (*f*t>MIC) were calculated graphically from the semilogarithmic plot of the concentration (total or free)-time data.
